# SEMPLR: an R package for transcription factor binding prediction

**DOI:** 10.1093/bioinformatics/btag383

**Published:** 2026-06-12

**Authors:** Grace E Kenney, Rintsen N Sherpa, Jeremy D Burgess, Alan P Boyle, Douglas H Phanstiel

**Affiliations:** Curriculum in Bioinformatics and Computational Biology, University of North Carolina, Chapel Hill, NC 27599, United States; Gilbert S. Omenn Department of Computational Medicine and Bioinformatics, University of Michigan, Ann Arbor, MI, United States; Thurston Arthritis Research Center, University of North Carolina, Chapel Hill, NC 27599, United States; Gilbert S. Omenn Department of Computational Medicine and Bioinformatics, University of Michigan, Ann Arbor, MI, United States; Department of Human Genetics, University of Michigan, Ann Arbor, MI United States; Curriculum in Bioinformatics and Computational Biology, University of North Carolina, Chapel Hill, NC 27599, United States; Thurston Arthritis Research Center, University of North Carolina, Chapel Hill, NC 27599, United States; Department of Cell Biology and Physiology, University of North Carolina, Chapel Hill, NC 27599, United States

## Abstract

**Summary:**

SEMPLR is an R package that predicts transcription factor binding and variant effects using SNP Effect Matrices (SEMs), providing efficient, genome-wide scoring, enrichment testing, and visualization tools for comprehensive analysis of regulatory sequences.

**Availability:**

Available on GitHub at https://github.com/grkenney/SEMPLR and on Bioconductor at https://bioconductor.org/packages/release/bioc/html/SEMPLR.html.

## 1 Introduction

Transcription factor (TF) binding to DNA orchestrates gene regulation and drives phenotypic diversity (ENCODE Project Consortium 2012, Lambert *et al*. 2018). Determining when and where TFs bind in the genome can provide insight into how DNA sequences encode biological outcomes. This understanding becomes particularly crucial for human disease research, as nearly 90% of disease-associated genetic variants fall within non-coding regions and are thought to act via alterations to TF binding affinity (Hindorff *et al*. 2009). Although techniques like ChIP-seq (Johnson *et al*. 2007) and CUT&RUN (Skene and Henikoff 2017) can map binding genome-wide, the sheer number of TFs, cell types, and biological conditions makes comprehensive experimental profiling infeasible.

To address this limitation, substantial effort has been invested in predicting TF binding based on sequence alone. While recent advances have introduced TF binding models with greater capacity to model complex sequence features, including flexible motif-based approaches (Steinhaus *et al*. 2022) and deep learning-based predictors (Alipanahi *et al*. 2015, Zhou and Troyanskaya 2015, Avsec *et al*. 2021, Jaganathan *et al*. 2025), matrix-based methods remain widely used due to their computational efficiency, interpretability, and compatibility with established genomic analysis workflows. Many of these existing matrix-based tools compare DNA sequences to position-weighted matrices (PWMs) which typically represent consensus binding motifs (Stormo 2000, Wanniarachchi *et al*. 2024), producing measurements of similarity to consensus motifs rather than quantitative predictions of actual binding likelihood under physiological conditions. To overcome this challenge, we previously developed the SNP Effect Matrix pipeline (SEMpl) (Nishizaki *et al*. 2020), a command-line tool that incorporates ChIP-seq and DNase-seq data to generate SNP Effect Matrices (SEMs). Unlike traditional PWMs, SEMs integrate chromatin accessibility information and empirical binding data to provide quantitative estimates of TF binding affinity that more accurately reflect binding. However, SEMpl’s only functionality is to build SEMs and report quality control metrics and lacks key features for research applications such as motif enrichment analysis, variant scoring, and integration with common bioinformatic frameworks.

Here, we introduce SEMPLR, an R package that extends the functionality of SEMpl by providing a comprehensive framework for SEM-based sequence analysis. SEMPLR includes optimized data structures for storing and manipulating SEMs, efficient algorithms for genome-wide scoring, and integrated visualization tools. It also provides methods for TF binding enrichment analysis and variant effect prediction, all tightly integrated within the Bioconductor ecosystem, enabling seamless incorporation into existing genomic analysis workflows and ensuring compatibility with standard data formats and analytical pipelines.

## 2 Summary

At its core, SEMPLR compares user-provided sequences or genomic regions to sets of SEMs to predict TF binding affinities. It also includes functions to predict how genetic variants alter TF binding, assess enrichment of TF binding sites in regions of interest, and visualize the results.

## 3 Implementation

SEMPLR is available as part of the Bioconductor ecosystem and was developed in R (v4.5). SEMPLR leverages existing Bioconductor data structures for seamless integration and compatibility with existing bioinformatic workflows, while also introducing new S4 data structures and accessor functions to easily contain, query, and manipulate SEM-specific data types.

SEMPLR calculates binding affinity scores for user-defined lists of DNA sequences or genomic ranges provided as GRanges or VRanges objects. A default set of 223 SEMs is included with SEMPLR, allowing users to conduct their entire analysis in R. If the provided set is not sufficient, a custom set can be generated with the SEMpl command line tool. Results are stored in a new S4 object, aggregating variant, SEM, and binding affinity scores for each sequence and SEM combination. Visualization functions operate directly on these results, producing ggplot objects, enabling easy plot customization.

## 4 Functionality

### 4.1 Predicting TF binding

**Figure 1:**
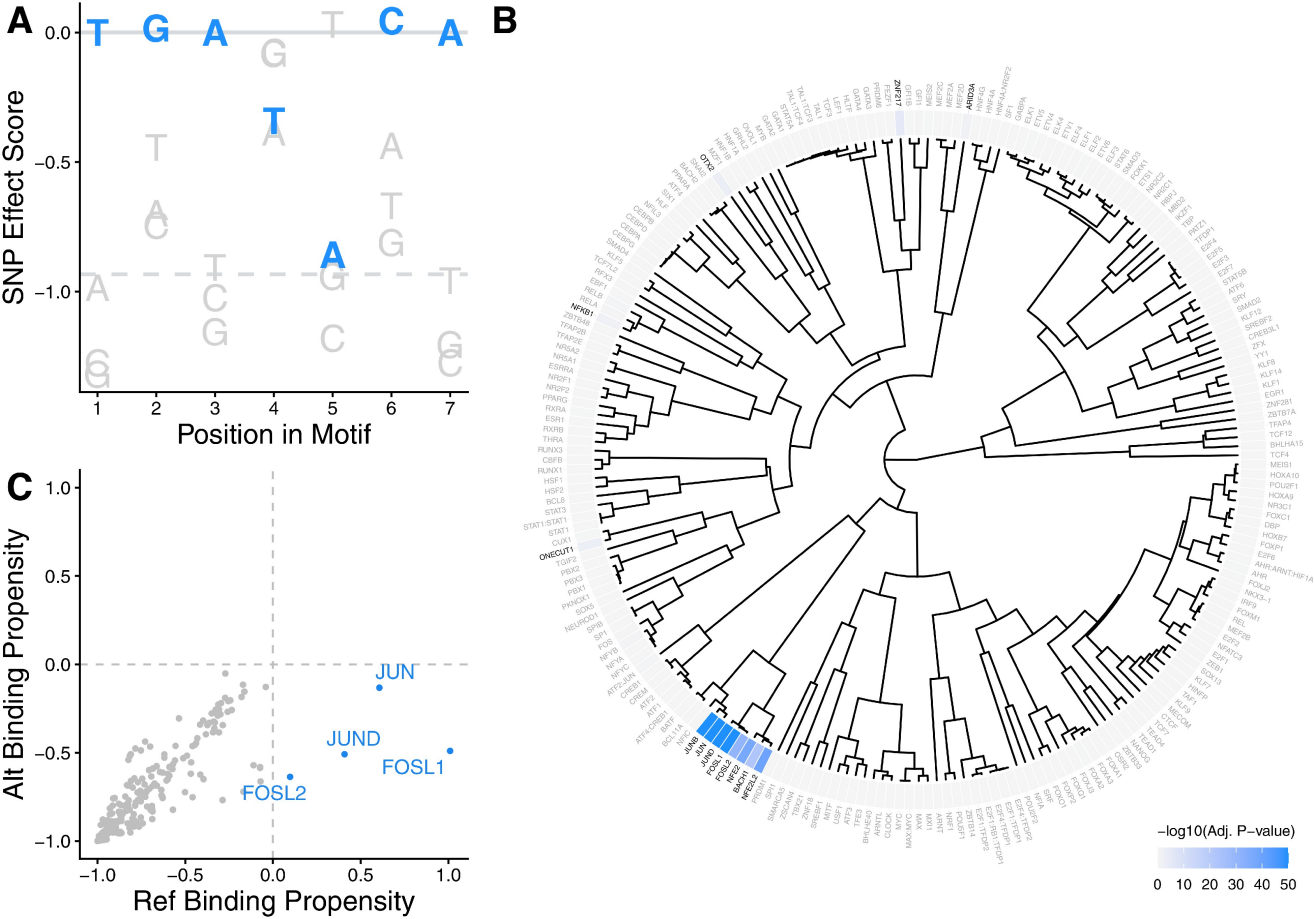
Examples of plots created using the *plotSEM*, *plotEnrich*, and *plotSEMMotifs* functions. (A) The plot created by the *plotSEM* function depicts the position-specific scores of each nucleotide of the SEM for the JUN motif (JASPAR MA0099.2), highlighting a query sequence. (B) The plot created by the *plotEnrich* function depicts the SEM enrichment scores across 1000 simulated sequences. 200 sequences were simulated from the JUN SEM, the remaining 800 sequences were randomly generated. TFs are hierarchically clustered by weighted Pearson correlation of SEMs; significantly enriched TFs (FDR < 0.01) appear in black font. (C) The plot created by the *plotSEMMotifs* function compares the allele-specific binding affinity scores for variant rs1357635 across the default sets of SEMs.

SEMPLR provides two core scoring functions. The first, *scoreBinding*, scores query sequences against each of the provided SEMs to generate binding affinity scores. These scores represent TF binding affinity at the highest-scoring site within each query sequence, normalized relative to background ChIP-seq signal. More positive scores indicate the TF is more likely to bind the query sequence, while more negative scores indicate the TF is less likely to bind. SEM scores for a query sequence can be visualized at single nucleotide resolution using the *plotSEM* function (Fig. 1A).

### 4.2 Predicting enrichment of TF binding

When analyzing large sets of genomic regions, it is often useful to determine whether binding by one or more TFs occurs more frequently than expected by chance. To address this, SEMPLR provides the *enrichSEMs* function, which performs a binomial test comparing binding scores for query regions to those from a scrambled or user-defined background. The function operates directly on SEMPLR scoring results and returns binding frequencies for both query and background sequences, along with *P*value for each SEM.

Motif enrichment tests often yield redundant results due to similarity among TF motifs. To address this, SEMPLR’s *plotEnrich* function leverages motif clustering functionality from universalmotif (Tremblay 2024) to calculate the weighted Pearson’s correlation coefficients between motifs and constructs a radial dendrogram that visually arranges TFs by motif similarity (Fig. 1B). Enrichment *P* values are displayed within the dendrogram structure to allow for the identification of enriched sets of closely related TFs.

One potential application of this enrichment is to predict the TFs responsible for differentially expressed genes between two biological conditions. To aid in this analysis, SEMPLR includes functions to test for the enrichment of predicted TF binding sites at subsets of genes. Users provide foreground gene sets of interest (e.g. upregulated genes) and background gene sets (e.g. all expressed genes). The *enrichmentSets* function extracts promoter regions that can be supplied to *enrichSEMs* for prediction of binding site enrichment in foreground sets. Significant enrichment of TF binding in the promoters of upregulated genes can provide mechanistic insight into gene expression changes.

### 4.3 Predicting the effect of genetic variation on TF binding

Determining the effect of single-nucleotide polymorphisms (SNPs), insertions, and deletions in non-coding regions of the genome is a critical step in evaluating the functional impact of genetic variation. Variants within TF binding sites are enriched among GWAS loci and may reveal causal regulatory mechanisms (ENCODE Project Consortium 2012; Lambert *et al*. 2018). While multiple tools can identify how genetic variants alter similarity to PWMs (Coetzee *et al*. 2015; Steinhaus *et al*. 2022; Zuo *et al*. 2015), no tools are available to predict changes in TF binding propensity using SEMs. SEMPLR aims to identify instances of genetic variants that alter TF binding, either breaking a TF motif sufficiently to prevent binding or strengthening a motif sufficiently to create a new binding event. This is accomplished through SEMPLR’s second scoring function, *scoreVariants*.

Using SEM scoring, users can predict how individual nucleotide changes alter TF binding affinity. The *scoreVariants* function accepts variants as VRanges or GRanges objects and computes allele-specific binding affinity scores for each SEM. As with the *scoreBinding* function, *scoreVariants* produces a *SEMScores* object aggregating variant, SEM, and scoring results. Variants with negative reference allele scores and positive alternative allele scores indicate potential gained binding events, while variants with positive reference allele scores and negative alternative allele scores indicate potential loss of binding events. These comparisons can be visualized in bulk on both the motif and variant levels with SEMPLR’s *plotSEMVariants* and *plotSEMMotifs* functions, respectively (Fig. 1C).

## 5 Benchmarking

In our previous work, we have demonstrated that SEM scoring more strongly correlates to ChIP-seq data compared to scores generated by PWMs and popular deep learning methods (Nishizaki *et al*. 2020). Here, we evaluated the ability of SEM-based scoring implemented in SEMPLR to classify TF binding events using ENCODE ATAC-seq and ChIP-seq data from K562 cells (ENCODE Project Consortium 2012; Table S1, available as supplementary data at *Bioinformatics* online). For each TF, ATAC-seq peaks overlapping ChIP-seq peaks were treated as true binding events, while ATAC-seq peaks lacking ChIP-seq overlap served as negatives. Binding affinity scores were computed using both SEMPLR and traditional PWM-based scoring using PWMs from HOCOMOCOv11 (Kulakovskiy *et al*. 2018; Table S2, available as supplementary data at *Bioinformatics* online). Classification performance was assessed using receiver operating characteristic (ROC) analysis.

Across 83 TFs with at least 500 ChIP-supported binding events, SEM-based scores consistently achieved higher ROC area-under-the-curve (AUC) values than PWM scores (paired Wilcoxon test, *P* = 1.56 × 10^−10^; Supplemental Fig 1A, available as supplementary data at *Bioinformatics* online). As an illustrative example, SEM-based scoring for RUNX1 produced substantially improved classification performance relative to PWM scoring (AUC 0.799 vs. 0.705; Supplemental Fig 1B, available as supplementary data at *Bioinformatics* online). Together, these results demonstrate that SEM-based scoring more accurately discriminates bound from unbound regulatory regions than PWM-based approaches across a broad set of TFs.

## 6 Conclusion

SEMPLR is an R package that extends transcription factor binding analysis beyond motif similarity to quantitative prediction of binding affinity and variant effects. By leveraging SNP Effect Matrices (SEMs) and integrating seamlessly within the Bioconductor ecosystem, SEMPLR enables efficient, genome-wide evaluation of TF binding, enrichment, and variant impact. This framework provides researchers with an accessible and reproducible approach for interpreting regulatory variation and advancing understanding of gene regulation in both basic and translational contexts.

## Supplementary Material

btag383_Supplementary_Data

## Data Availability

Source code and documentation for SEMPLR are available on GitHub (https://github.com/grkenney/SEMPLR). SEMPLR can be installed via Bioconductor (https://bioconductor.org/packages/SEMPLR). ATAC data used for benchmarking was downloaded from ENCODE and PWMs were downloaded from HOCOMOCOv11. Accession numbers for these data are listed in the online supplementary material.
