# Supplementary material for: SEMPLR: an R package for transcription factor binding prediction
Source: Bioinformatics. 2026 Jun 12;42(6):btag383. doi: 10.1093/bioinformatics/btag383 (PMC13294450; doi:10.1093/bioinformatics/btag383)
Supplement: btag383_Supplementary_Data [file btag383_supplementary_data.zip › Supplementary_Tables_and_Figure.pdf]

Supplemental Table 1. ENCODE datasets used in benchmarking analysis
List of ENCODE files used for SEMPLR benchmarking against PWM scoring (Supplemental Figure 1).

Experiment	Bed file	Target	Cell line
ENCSR483RKN	ENCFF558BLC	ATAC	Homo sapiens K562
ENCSR613NUC	ENCFF553BVI	ARNT	Homo sapiens K562
ENCSR000DNZ	ENCFF206BGR	ATF1	Homo sapiens K562
ENCSR014ARU	ENCFF121HYT	ATF2	Homo sapiens K562
ENCSR568ZXG	ENCFF246USC	ATF3	Homo sapiens K562
ENCSR044UJJ	ENCFF250MUC	ATF4	Homo sapiens K562
ENCSR125RFR	ENCFF863ZFH	ATF6	Homo sapiens K562
ENCSR740NPG	ENCFF678TXX	BACH1	Homo sapiens K562
ENCSR802AHH	ENCFF941EDY	BCL6	Homo sapiens K562
ENCSR000EGV	ENCFF154IVU	BHLHE40	Homo sapiens K562
ENCSR116NDV	ENCFF802NHC	CBFB	Homo sapiens K562
ENCSR000BRQ	ENCFF022KBK	CEBPB	Homo sapiens K562
ENCSR058DRG	ENCFF558AJI	CEBPG	Homo sapiens K562
ENCSR228OQM	ENCFF648UJW	CLOCK	Homo sapiens K562
ENCSR471WXT	ENCFF970QKS	CREB1	Homo sapiens K562
ENCSR077DKV	ENCFF324ELP	CREM	Homo sapiens K562
ENCSR000EGM	ENCFF396BZQ	CTCF	Homo sapiens K562
ENCSR000EFO	ENCFF838GFC	CUX1	Homo sapiens K562
ENCSR153DWR	ENCFF904RTN	E2F1	Homo sapiens K562
ENCSR036QIR	ENCFF710UAZ	E2F3	Homo sapiens K562
ENCSR368GJN	ENCFF221TRR	E2F4	Homo sapiens K562
ENCSR744GHR	ENCFF173QUY	E2F5	Homo sapiens K562
ENCSR000BLI	ENCFF831NDQ	E2F6	Homo sapiens K562
ENCSR171CAY	ENCFF938ZPZ	E2F7	Homo sapiens K562

ENCSR744CXJ	ENCFF763MXW	ELF1	Homo sapiens K562
ENCSR594HXD	ENCFF695PDY	ELF2	Homo sapiens K562
ENCSR486IFJ	ENCFF588QRH	ESRRA	Homo sapiens K562
ENCSR000BKQ	ENCFF886BDQ	ETS1	Homo sapiens K562
ENCSR277DMR	ENCFF065RZP	ETV1	Homo sapiens K562
ENCSR971EWR	ENCFF604LXR	ETV5	Homo sapiens K562
ENCSR000BMV	ENCFF004HXL	FOSL1	Homo sapiens K562
ENCSR231ZVN	ENCFF504WWL	FOXA3	Homo sapiens K562
ENCSR056VMR	ENCFF567CPM	FOXJ3	Homo sapiens K562
ENCSR659CCI	ENCFF516ZWP	FOXK1	Homo sapiens K562
ENCSR438RRM	ENCFF820MJD	FOXM1	Homo sapiens K562
ENCSR530WIV	ENCFF491EEI	FOXP1	Homo sapiens K562
ENCSR290MUH	ENCFF889XXJ	GABPA	Homo sapiens K562
ENCSR948VFL	ENCFF637SIR	IKZF1	Homo sapiens K562
ENCSR926KTP	ENCFF724CHN	IRF9	Homo sapiens K562
ENCSR525VAT	ENCFF932KRN	JUNB	Homo sapiens K562
ENCSR000DJX	ENCFF273KIA	JUND	Homo sapiens K562
ENCSR550HCT	ENCFF674KVR	KLF1	Homo sapiens K562
ENCSR343ELW	ENCFF591TLJ	LEF1	Homo sapiens K562
ENCSR158LJN	ENCFF266YHW	MAX	Homo sapiens K562
ENCSR221GAN	ENCFF788YHU	MBD2	Homo sapiens K562
ENCSR935UQL	ENCFF188PLS	MECOM	Homo sapiens K562
ENCSR647ZXA	ENCFF242ULW	MEF2D	Homo sapiens K562
ENCSR851BNE	ENCFF861ZJL	MEIS2	Homo sapiens K562
ENCSR797SWM	ENCFF076GSK	MITF	Homo sapiens K562
ENCSR000EGZ	ENCFF068IGH	MXI1	Homo sapiens K562
ENCSR986CDX	ENCFF625QHR	NEUROD1	Homo sapiens K562

ENCSR670FDA	ENCFF082EPO	NFATC3	Homo sapiens K562
ENCSR000FCC	ENCFF474PRJ	NFE2	Homo sapiens K562
ENCSR796ITY	ENCFF092TVM	NFIC	Homo sapiens K562
ENCSR163VTS	ENCFF786CRW	NFYA	Homo sapiens K562
ENCSR742IDN	ENCFF469ZBB	NR2C1	Homo sapiens K562
ENCSR516SWI	ENCFF946CTR	NR2C2	Homo sapiens K562
ENCSR970NKQ	ENCFF746GDG	NR2F1	Homo sapiens K562
ENCSR000BRS	ENCFF847ZHF	NR2F2	Homo sapiens K562
ENCSR980ZWE	ENCFF201BGD	NR3C1	Homo sapiens K562
ENCSR494TDU	ENCFF543STN	NRF1	Homo sapiens K562
ENCSR633EIC	ENCFF925OBR	PBX2	Homo sapiens K562
ENCSR115SMW	ENCFF099RDJ	PKNOX1	Homo sapiens K562
ENCSR364SNE	ENCFF724DNU	POU5F1	Homo sapiens K562
ENCSR740XMB	ENCFF691JZW	RBPJ	Homo sapiens K562
ENCSR414TYY	ENCFF374EFU	RUNX1	Homo sapiens K562
ENCSR376XAV	ENCFF718DSJ	SMAD3	Homo sapiens K562
ENCSR068QXO	ENCFF934NEX	SMAD4	Homo sapiens K562
ENCSR895HSJ	ENCFF722RWS	SMARCA5	Homo sapiens K562
ENCSR000BGW	ENCFF888CKG	SPI1	Homo sapiens K562
ENCSR815ZDS	ENCFF029RBI	SREBF1	Homo sapiens K562
ENCSR582IAO	ENCFF087EVW	SRF	Homo sapiens K562
ENCSR338UQU	ENCFF598VGY	STAT6	Homo sapiens K562
ENCSR744WOO	ENCFF808QUD	TCF12	Homo sapiens K562
ENCSR970OJY	ENCFF773RNU	TCF3	Homo sapiens K562
ENCSR863KUB	ENCFF512IAI	TCF7	Homo sapiens K562
ENCSR888XZK	ENCFF732ZNB	TCF7L2	Homo sapiens K562
ENCSR591ASD	ENCFF692HUD	TEAD1	Homo sapiens K562

ENCSR901VIE	ENCFF044IDV	TFDP1	Homo sapiens K562
ENCSR953KEY	ENCFF592NJN	TFE3	Homo sapiens K562
ENCSR264CZJ	ENCFF309DMZ	THRA	Homo sapiens K562
ENCSR787CHF	ENCFF255QDL	USF1	Homo sapiens K562
ENCSR628DJK	ENCFF840NZE	ZFX	Homo sapiens K562
ENCSR214EKV	ENCFF648ORA	ZNF281	Homo sapiens K562

Supplemental Table 2. HOCOMOCOv11 PWMs used in benchmarking analysis

List of HOCOMOCOv11 files used for SEMPLR benchmarking against PWM scoring (Supplemental Figure 1).

**Transcription Factor**	**PWM**
ARNT	ARNT_HUMAN.H11MO.0.B
ATF1	ATF1_HUMAN.H11MO.0.B
ATF2	ATF2_HUMAN.H11MO.0.B
ATF3	ATF3_HUMAN.H11MO.0.A
ATF4	ATF4_HUMAN.H11MO.0.A
ATF6	ATF6A_HUMAN.H11MO.0.B
BACH1	BACH1_HUMAN.H11MO.0.A
BCL6	BCL6_HUMAN.H11MO.0.A
BHLHE40	BHE40_HUMAN.H11MO.0.A
CBFB	PEBB_HUMAN.H11MO.0.C
CEBPB	CEBPB_HUMAN.H11MO.0.A
CEBPG	CEBPG_HUMAN.H11MO.0.B
CLOCK	CLOCK_HUMAN.H11MO.0.C
CREB1	CREB1_HUMAN.H11MO.0.A
CREM	CREM_HUMAN.H11MO.0.C
CTCF	CTCF_HUMAN.H11MO.0.A
CUX1	CUX1_HUMAN.H11MO.0.C
E2F1	E2F1_HUMAN.H11MO.0.A
E2F3	E2F3_HUMAN.H11MO.0.A
E2F4	E2F4_HUMAN.H11MO.0.A
E2F5	E2F5_HUMAN.H11MO.0.B
E2F6	E2F6_HUMAN.H11MO.0.A
E2F7	E2F7_HUMAN.H11MO.0.B
ELF1	ELF1_HUMAN.H11MO.0.A

ELF2	ELF2_HUMAN.H11MO.0.C
ESRRA	ERR1_HUMAN.H11MO.0.A
ETS1	ETS1_HUMAN.H11MO.0.A
ETV1	ETV1_HUMAN.H11MO.0.A
ETV5	ETV5_HUMAN.H11MO.0.C
FOSL1	FOSL1_HUMAN.H11MO.0.A
FOXA3	FOXA3_HUMAN.H11MO.0.B
FOXJ3	FOXJ3_HUMAN.H11MO.0.A
FOXK1	FOXK1_HUMAN.H11MO.0.A
FOXM1	FOXM1_HUMAN.H11MO.0.A
FOXP1	FOXP1_HUMAN.H11MO.0.A
GABPA	GABPA_HUMAN.H11MO.0.A
IKZF1	IKZF1_HUMAN.H11MO.0.C
IRF9	IRF9_HUMAN.H11MO.0.C
JUNB	JUNB_HUMAN.H11MO.0.A
JUND	JUND_HUMAN.H11MO.0.A
KLF1	KLF1_HUMAN.H11MO.0.A
LEF1	LEF1_HUMAN.H11MO.0.A
MAX	MAX_HUMAN.H11MO.0.A
MBD2	MBD2_HUMAN.H11MO.0.B
MECOM	EVI1_HUMAN.H11MO.0.B
MEF2D	MEF2D_HUMAN.H11MO.0.A
MEIS2	MEIS2_HUMAN.H11MO.0.B
MITF	MITF_HUMAN.H11MO.0.A
MXI1	MXI1_HUMAN.H11MO.0.A
NEUROD1	NDF1_HUMAN.H11MO.0.A
NFATC3	NFAC3_HUMAN.H11MO.0.B

NFE2	NFE2_HUMAN.H11MO.0.A
NFIC	NFIC_HUMAN.H11MO.0.A
NFYA	NFYA_HUMAN.H11MO.0.A
NR2C1	NR2C1_HUMAN.H11MO.0.C
NR2C2	NR2C2_HUMAN.H11MO.0.B
NR2F1	COT1_HUMAN.H11MO.0.C
NR2F2	COT2_HUMAN.H11MO.0.A
NR3C1	GCR_HUMAN.H11MO.0.A
NRF1	NRF1_HUMAN.H11MO.0.A
PBX2	PBX2_HUMAN.H11MO.0.C
PKNOX1	PKNX1_HUMAN.H11MO.0.B
POU5F1	PO5F1_HUMAN.H11MO.0.A
RBPJ	SUH_HUMAN.H11MO.0.A
RUNX1	RUNX1_HUMAN.H11MO.0.A
SMAD3	SMAD3_HUMAN.H11MO.0.B
SMAD4	SMAD4_HUMAN.H11MO.0.B
SMARCA5	SMCA5_HUMAN.H11MO.0.C
SPI1	SPI1_HUMAN.H11MO.0.A
SREBF1	SRBP1_HUMAN.H11MO.0.A
SRF	SRF_HUMAN.H11MO.0.A
STAT6	STAT6_HUMAN.H11MO.0.B
TCF12	HTF4_HUMAN.H11MO.0.A
TCF3	TFE2_HUMAN.H11MO.0.A
TCF7	TCF7_HUMAN.H11MO.0.A
TCF7L2	TF7L2_HUMAN.H11MO.0.A
TEAD1	TEAD1_HUMAN.H11MO.0.A
TFDP1	TFDP1_HUMAN.H11MO.0.C

TFE3	TFE3_HUMAN.H11MO.0.B
THRA	THA_HUMAN.H11MO.0.C
USF1	USF1_HUMAN.H11MO.0.A
ZFX	ZFX_HUMAN.H11MO.0.A
ZNF281	ZN281_HUMAN.H11MO.0.A

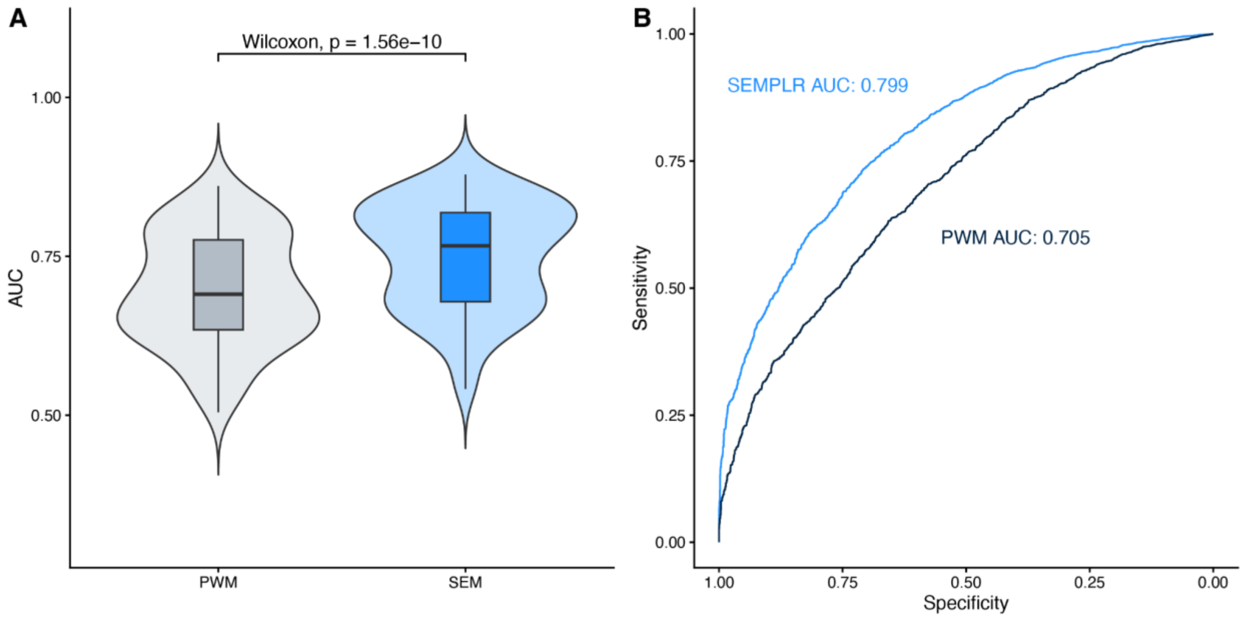

Supplemental Figure 1.

SEM scoring better predicts ChIP binding compared to PWM scoring. (A) PWM and SEM scores for 83 motifs were computed in a random sample of 50,000 ATAC peaks (Human K562 cells, ENCODE accession: ENCFF558BLC) using corresponding PWMs from HOCOMOCOv11 and SEMs from the SEMPLR's default set. The ability of these scores to predict true binding was evaluated with a receiver operating characteristic (ROC) curve where true binding was defined by overlapping the scored ATAC peaks with ChIP-seq peaks for the respective TF (Supplemental Table 1). SEM scoring with SEMPLR resulted in significantly higher area under the curves (AUCs) than PWM scoring (paired Wilcoxon p = 1.56×10^{-10}), indicating a better classification of binding events. (B) A representative example of the ROC curve for a single TF, RUNX1.
