# Supplementary figures and images for: SEMPLR: an R package for transcription factor binding prediction
Source: Bioinformatics. 2026 Jun 12;42(6):btag383. doi: 10.1093/bioinformatics/btag383 (PMC13294450; doi:10.1093/bioinformatics/btag383)

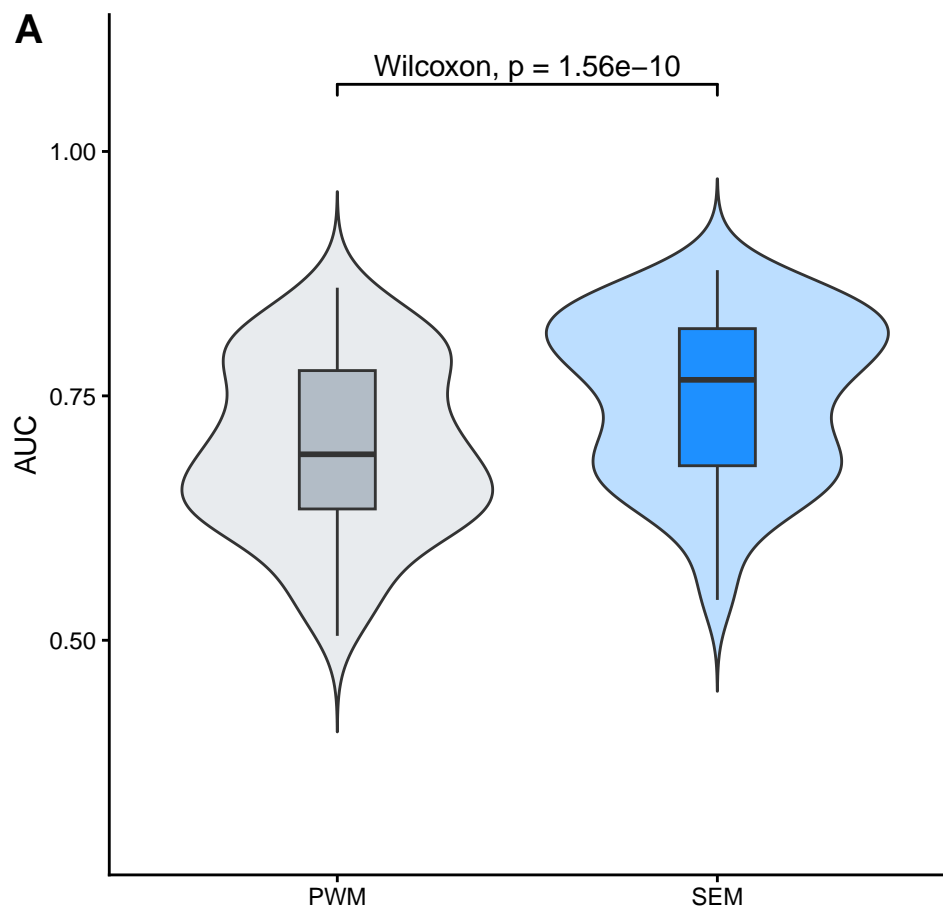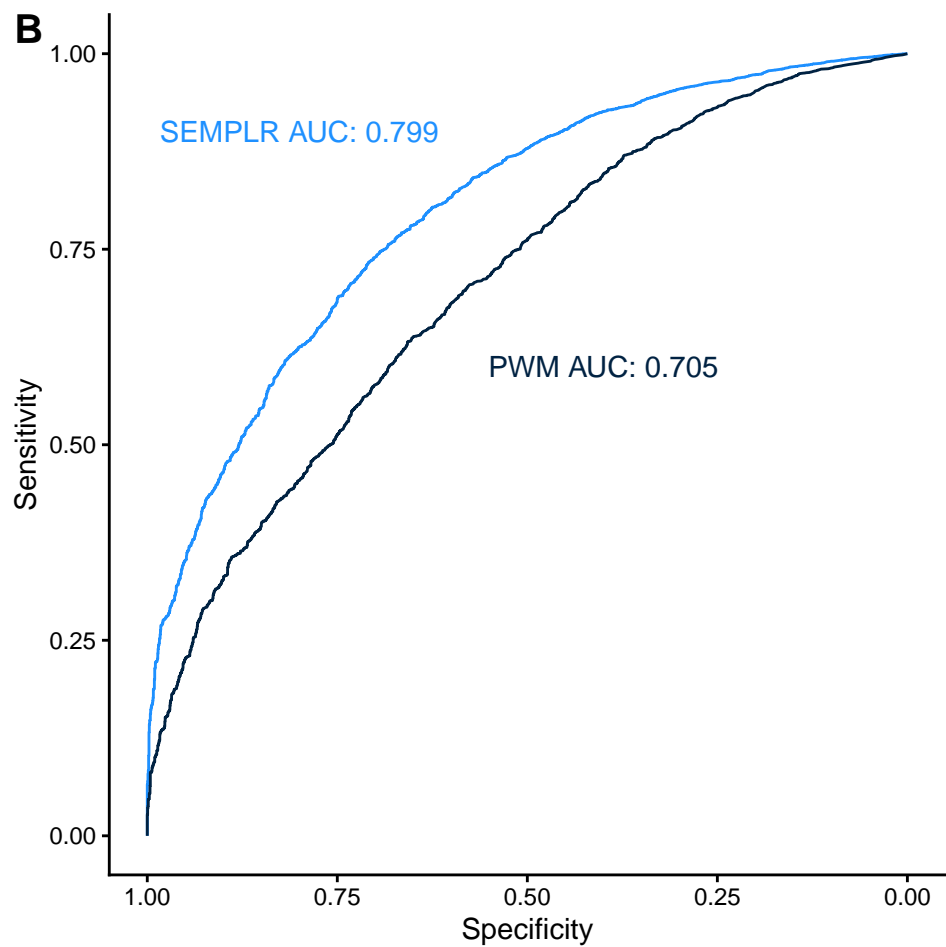

Supplement: btag383_Supplementary_Data [file btag383_supplementary_data.zip › Supplemental_Figure.pdf]
